# Supplementary figures and images for: Efficacy of the therapeutic use of video games on the depressive state of stroke patients: Protocol for systematic review and meta-analysis
Source: PLoS One. 2022 Dec 28;17(12):e0275740. doi: 10.1371/journal.pone.0275740 (PMC9797084; doi:10.1371/journal.pone.0275740)

**Figure 1**. Plan of study screening and selection process


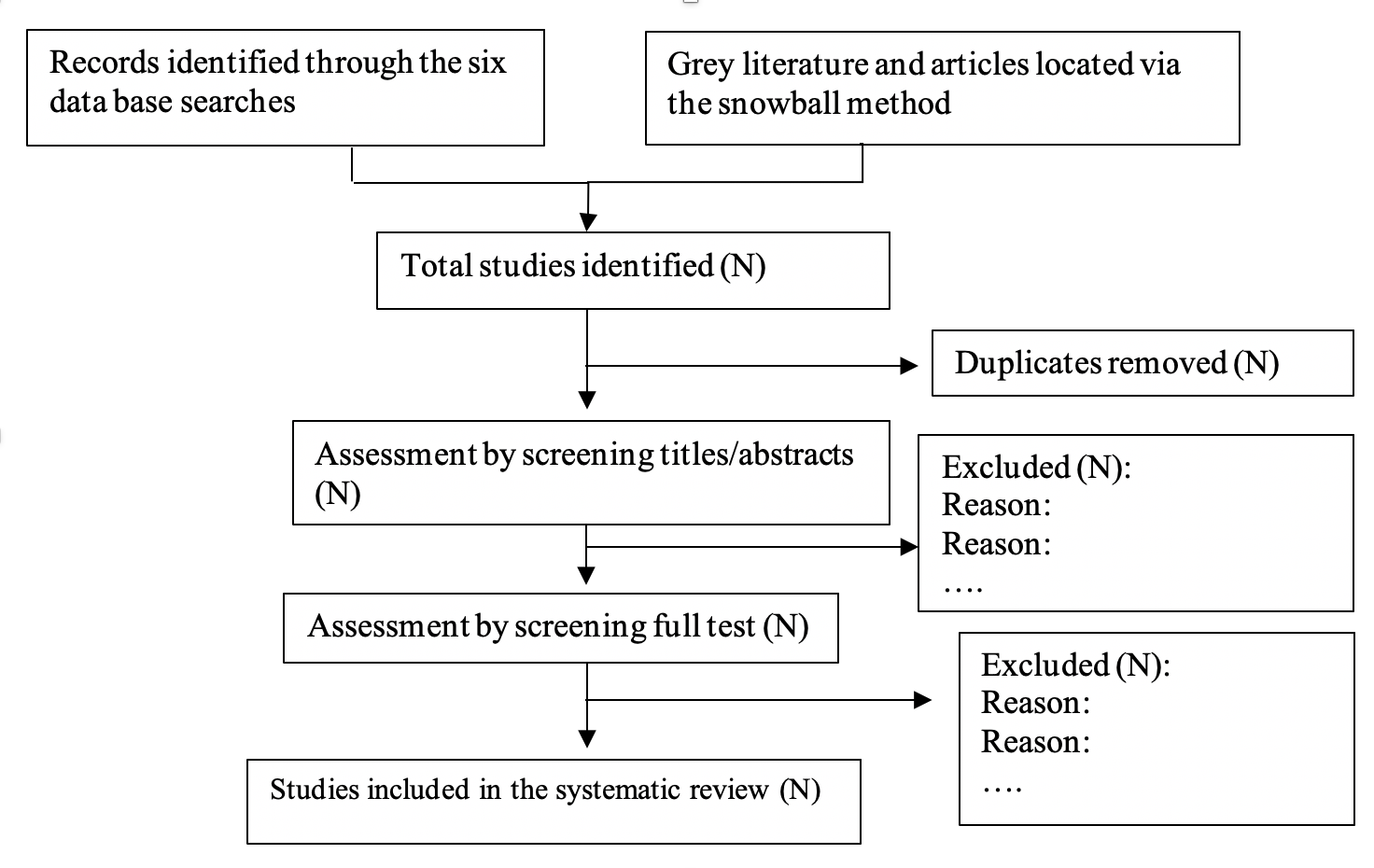

Supplement: S1 Fig — (DOCX) [file pone.0275740.s001.docx]
