# Supplementary material for: Efficacy of the therapeutic use of video games on the depressive state of stroke patients: Protocol for systematic review and meta-analysis
Source: PLoS One. 2022 Dec 28;17(12):e0275740. doi: 10.1371/journal.pone.0275740 (PMC9797084; doi:10.1371/journal.pone.0275740)
Supplement: S2 Appendix — (DOCX) [file pone.0275740.s003.docx]

| **Database**  S 2 Appendix. The search strategy. | **Descriptors** | **Fiiters** |
| --- | --- | --- |
| PubMed | ((((((((((((((((("Stroke"[Mesh]) OR "Hemorrhagic Stroke"[Mesh]) OR "Embolic Stroke"[Mesh]) OR "Ischemic Stroke"[Mesh]) OR ( "Stroke, Lacunar"[Mesh] OR "Thrombotic Stroke"[Mesh] )) OR "Infarction, Posterior Cerebral Artery"[Mesh]) OR "Brain Stem Infarctions"[Mesh]) OR "Infarction, Middle Cerebral Artery"[Mesh]) OR "Infarction, Anterior Cerebral Artery"[Mesh]) AND "Virtual Reality"[Mesh]) OR "Virtual Reality Exposure Therapy"[Mesh]) OR "Exergaming"[Mesh]) | Clinicals trials |
| Cochrane  23 Potential | #1 MeSH descriptor: [Stroke] explode all trees  #2 MeSH descriptor: [Virtual Reality] explode all trees  #3 MeSH descriptor: [Rehabilitation] explode all trees  #4 #1 AND #2 AND 3# | Trials |
| Cinahl | stroke or cerebrovascular accident or cva ) AND ( virtual reality or vr or augmented reality ) AND clinical trial |  |
| PsycInfo | ( stroke or cerebrovascular accident or cva ) AND ( virtual reality or vr or augmented reality ) | Filtro clinical trial |
| Embase | ((**stroke**:ti OR **'hemorragic stroke'**:ti OR **'embolic stroke'**:au OR **'ischemic stroke'**:ti OR **'stroke, lacular'**:ti OR **'thrombotic stroke'**:ti OR **'infarction,posterior cerebral artery'**:ti OR **'brain stem infarctions'**:ti OR **'infarction, middle cerebral artery'**:ti OR **'infarction, anterior cerebral artery'**:ti) AND **'virtual reality'**:ti OR **'virtual reality exposure therapy'**:ti OR **exergaming**:ti) AND ([controlled clinical trial]/lim OR [randomized controlled trial]/lim) | Controlled Clinical trial  Randomized controlled trial |
| WOS | TI = (Stroke AND Virtual reality brain) OR  TI=(Stroke and virtual therapy) or TI = ( brain infarction and virtual therapy) |  |
| Grey literature | **Grey literature databases:**  ACM DL Digital Library  Conference Proceedings Citation Index  Open Grey |  |
