# Supplementary material for: Efficacy of the therapeutic use of video games on the depressive state of stroke patients: Protocol for systematic review and meta-analysis
Source: PLoS One. 2022 Dec 28;17(12):e0275740. doi: 10.1371/journal.pone.0275740 (PMC9797084; doi:10.1371/journal.pone.0275740)
Supplement: Attachment — Submitted filename: Response to reviewers_R1.docx [file pone.0275740.s004.docx]

**Response to Editor and Reviewers:**

Thank you very much for all your recommendations and suggestions. They have served as a guide to improve the quality of the manuscript and we have tried to follow them with maximum rigour. We would like to thank the reviewers for their expeditious and helpful comments. We have made every effort to answer all their questions and have modified the manuscript according to their suggestions. What follows is a description of all changes made. The revised version of the manuscript has all changes in yellow and our responses to the points you have raised are outlined below in a table.

| **Editor** | **Response to comments** |
| --- | --- |
| After careful consideration, we feel that it has merit but does not fully meet PLOS ONE’s publication criteria as it currently stands. Therefore, we invite you to submit a revised version of the manuscript that addresses the points raised during the review process. | Dear Editor and reviewers,  We appreciate the opportunity and invitation to resubmit our revised manuscript, after addressing all reviewers’ comments. We would also like to thank the reviewer for the suggestions and comments, which have undoubtedly improved the quality of our manuscript.  We have carefully considered all issues mentioned in reviewers' comments, outlined every change made point by point, and provided suitable rebuttals for any comments not addressed.  Finally, we have highlighted in yellow all changes made in the revised manuscript.  Taking the opportunity to make changes, we have updated some of the author affiliations. |

| **Reviewer #1** | **Response to comments** |
| --- | --- |
| “The high morbidity and mortality rates associated with have led to the increasing creation of specialized units for its treatment.” The sentence lacks a word. I think “stroke”. | Thank you very much for recommendation. We have included the word Stroke in the sentence as you have indicated. |
| “Studies comparing two different types of virtual reality without an alternative group will be not include”. Included not include. | Thank you for your recommendation. We have better described the criterion by modifying the sentence and verb tense. |
| However, a revision by a native speaker is definitely needed. In the present form the article is not suitable for publication. | Thank you for your recommendation. The article has been reviewed and proofread by a native speaker. |
| A recent review extensively discusses the use of digital therapeutics in the management of patients with chronic neurological disorders, with a specific focus on VR (PMID: 34018047). A further recent article summarize the application of telemedicine in stroke patients’ management (PMID: 33433756). They deserve to be mentioned. | Thank you for your recommendation. We have included these and other references, as well as reviewed the rest of the references. |
| In the “data sources and search strategy” section, the verb tenses are not congruent. Please, revised it. | Thank you for your recommendation. The verb tenses have been modified, as they were incorrect. |
| List exclusion criteria and inclusion criteria that so scattered are not immediate. | Thank you for your recommendation. We have listed the inclusion criteria for ease of understanding. |
| Conclusions should be better related to the purpose of the review. | Thank you for your recommendation. We have expanded on the discussion by focusing on the objectives of the study. |
